# Workflow of the zero‐fluoro pulsed field ablation

**DOI:** 10.1002/joa3.13174

**Published:** 2024-10-21

**Authors:** Shu Hirata, Koichi Nagashima, Ryuta Watanabe, Yuji Wakamatsu, Moyuru Hirata, Sayaka Kurokawa, Naoto Otsuka, Masanaru Sawada, Yasuo Okumura

**Affiliations:** ^1^ Division of Cardiology, Department of Medicine Nihon University School of Medicine Tokyo Japan

**Keywords:** atrial fibrillation, catheter ablation, pulmonary vein, pulsed field ablation

## Abstract

**Background:**

Zero‐fluoroscopic pulsed field ablation (PFA) is the next step in advancing pulmonary vein isolation (PVI) for atrial fibrillation (AF).

**Methods:**

A workflow incorporating a variable‐loop circular PFA catheter, intracardiac echocardiography (ICE), and a visualized sheath was applied to 15 paroxysmal AF patients.

**Results:**

It consisted of three steps: (1) positioning a non‐magnetic mapping catheter in the coronary sinus after creating a right atrial matrix, (2) transseptal puncture with the sheath advancement to the left atrium under ICE guidance, and (3) PFA confirming catheter‐tissue contact with tissue proximity indication.

**Conclusions:**

This zero‐fluoro PFA workflow is feasible without advanced technique.

## Graphical abstract

The workflow for zero‐fluoroscopy pulsed field ablation (PFA) incorporates a variable‐loop circular PFA catheter, intracardiac echocardiography, and a deflectable sheath visualized by a 3‐dimensional mapping system. It consists of three main steps: (1) placing the nonmagnetic catheter in the coronary sinus, (2) performing the transseptal puncture using a deflectable sheath, and (3) conducting PFA guided by tissue proximity indication.

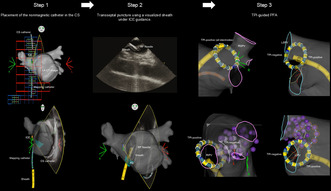

Recently, pulmonary vein isolation (PVI) using pulsed field ablation (PFA) for atrial fibrillation (AF) has become widely adopted, offering favorable efficacy and safety outcomes.^1^, ^2^ However, considerable fluoroscopy time is still used, posing a risk of malignant disease for both patients and operators.^3^ Novel technologies such as 3‐dimensional electro‐anatomical mapping (3D‐EAM) systems and intracardiac echocardiography (ICE) have been widely used to reduce radiation exposure, enabling the integration of electrical and anatomical information.^4^, ^5^, ^6^ In the current situation, the next step would be zero fluoroscopy PFA with the integration of a variable‐loop circular PFA catheter (VARIPULSE, Biosense Webster, Inc.; Irvine, CA), an ICE integration module (CARTOSOUND, Biosense Webster), and a deflectable sheath (VIZIGO, Biosense Webster) visualized by 3D‐EAM (CARTO3, Biosense Webster).^6^

Consecutive 15 paroxysmal AF patients (62 ± 10 years, 11 men) underwent PVI with PFA under deep sedation with dexmedetomidine, midazolam, and fentanyl. Through the femoral venous access, ICE catheter (SOUNDSTAR, Biosense Webster) and VIZIGO sheath were introduced. After creating a matrix of the entire right atrium (RA) with a magnetic sensor‐equipped mapping catheter (DECANAV, Biosense Webster), nonmagnetic catheters and the VIZIGO sheath can be visualized on 3D‐EAM. A 20‐pole electrode catheter (BeeAT, Japan Lifeline Co., Ltd.) was inserted into the CS via right jugular vein access on 3D‐EAM (Figure 1A).

**FIGURE 1:**
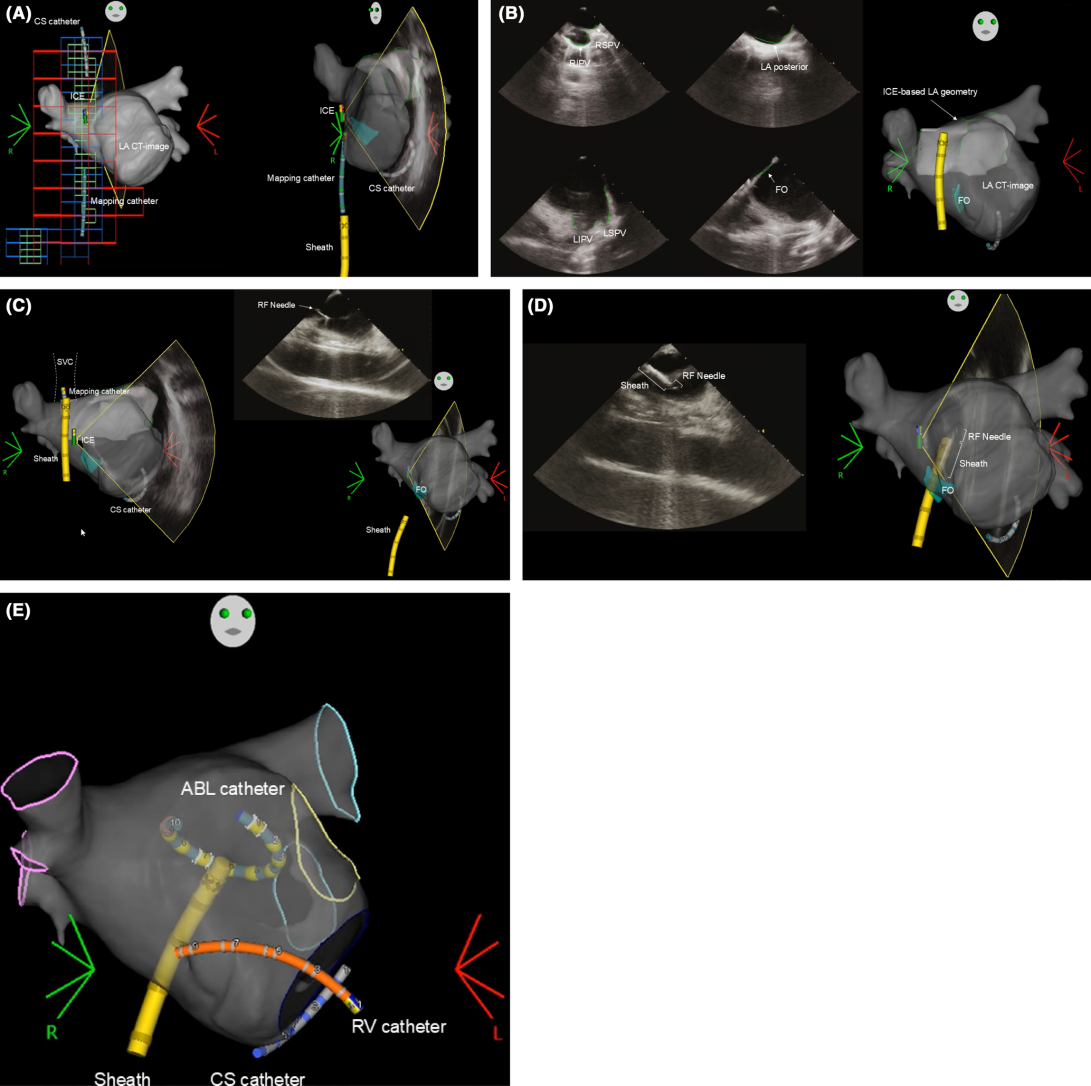
(A) Placement of the mapping catheter without a magnetic sensor. (B) Reconstruction of the geometry of the pulmonary veins (PVs), left atrium (LA) and fossa ovalis (FO). (C) Transseptal puncture with a the VIZIGO sheath under intracardiac echocardiography (ICE) guidance. (D) Advancement of the VIZIGO sheath into the LA. (E) Positioning the VARIPULSE catheter in the LA. CS, coronary sinus; CT, computed tomography; FO, fossa ovalis; ICE, intracardiac echocardiography; LA, left atrium; LIPV, left inferior pulmonary vein; LSPV, left superior pulmonary vein; RF, radiofrequency; RIPV, right inferior pulmonary vein; RSPV, right superior pulmonary vein; SVC, superior vena cava.

Simultaneously, the 3D geometry of the PVs, left atrium (LA) and fossa ovalis (FO) was reconstructed with the CARTOSOUND Module (Biosense Webster) and merged with the LA computed tomography images (Figure 1B). After positioning the DECANAV catheter and VIZIGO sheath into the superior vena cava in 3D‐EAM, the transseptal puncture was performed with a transseptal needle (RF Needle, Japan Lifeline Co., Ltd.) introduced through the VIZIGO sheath under ICE guidance (Figure 1C). Once the VIZIGO sheath was advanced into the LA through the FO on 3D‐EAM system (Figure 1D), the OCTARAY catheter (Biosense Webster) was introduced to the LA and a voltage/activation map was created.

By exchanging to the VARIPULSE catheter (Figure 1E), a base impedance map was built through a process in which impedance and location measurements are accumulated as the catheter is maneuvered in the LA for the tissue proximity indication (TPI). Under these conditions, once the electrode touches the PV/LA endocardium, a rapid change in impedance of 7% from the baseline illuminates an electrode as TPI‐positive on 3D‐EAM.^7^ PVI was performed according to the standard workflow recommendations, using the maximum energy setting to apply at least 12 applications per PV (6 applications to the distal PV with a rotation after the first 3 applications, followed by a second set of 6 applications to the proximal PV; Figure 2).^2^ At this time, DECANAV was advanced into the RV under 3D‐EAM guidance as backup pacing for temporary bradycardia caused by the reaction of the ganglionated plexi to PFA. For the VARIPULSE catheter‐tissue contact, the operators made an effort to ensure TPI‐positivity in all ablation electrodes. All PVs were isolated in all patients. At the end of the procedure, the absence of pericardial effusion was confirmed by ICE. The details of the ablation procedure, including the time from puncture to transseptal puncture, LA mapping time, PVI time, fluoroscopy time, the number of PF applications, and any complications, are shown in the Table 1.

**FIGURE 2:**
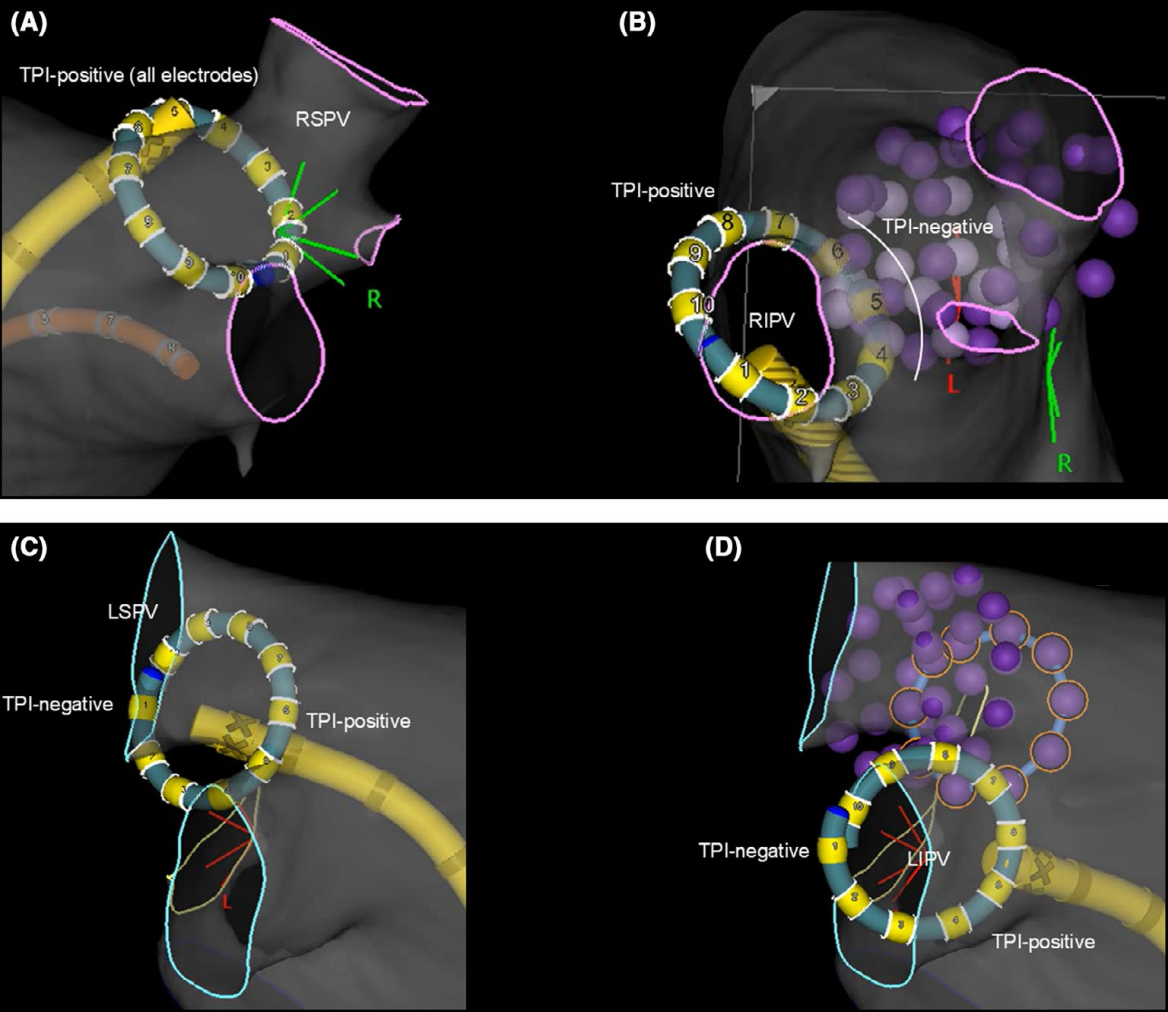
Pulsed field ablation with the VARIPULSE catheter for (A) right superior pulmonary vein (RSPV), (B) right inferior pulmonary vein (RIPV), (C) left superior pulmonary vein (LSPV), and (D) left inferior pulmonary vein (LIPV). An electrode with both sides lit up indicates a TPI positive. The dark purple tag indicates the sites where all three applications of pulse field energy were completed in each position. The light purple tag indicates the sites where only one or two applications were completed. The TPI filter is not used. LIPV, left inferior pulmonary vein; LSPV, left superior pulmonary vein; PVI, pulmonary vein isolation; RF, radiofrequency; RIPV, right inferior pulmonary vein; RSPV, right superior pulmonary vein; TPI, tissue proximity indication.

**TABLE 1:** Summary of patient characteristics and procedural data.

Case number	Age (years)	Sex	Time from puncture to atrial septal puncture (min)	Left atrial mapping time (min)	PVI time (min)	Total fluoroscopy dose (mGy)	Total number of PF applications	Complications
1	69	Male	26	8	24	0	59	None
2	53	Male	20	7	32	0	47	None
3	64	Female	14	9	27	0	54	None
4	68	Male	14	8	23	0	48	None
5	64	Female	15	7	30	0	48	None
6	50	Male	14	12	24	0	51	None
7	61	Male	15	8	18	0	48	None
8	50	Male	13	8	26	0	50	None
9	69	Male	16	11	16	0	51	None
10	57	Male	25	6	29	0	67	None
11	70	Male	15	7	25	0	49	None
12	58	Male	18	7	42	0	58	None
13	77	Female	20	10	34	0	63	None
14	77	Female	16	10	36	0	62	None
15	45	Male	20	11	25	0	48	None
	62 ± 10	Male (73%)	17.4 ± 4.0	8.7 ± 1.8	27.4 ± 6.8	0	53.5 ± 6.6	0

*Note*: Values are mean ± SD or *n* (%).

Abbreviations: AF, atrial fibrillation; PF, pulsed field; PVI, pulmonary vein isolation.

This zero‐fluoro PFA workflow consists of 3 steps; (1) placement of the nonmagnetic catheter in the CS, (2) the transseptal puncture by VIZIGO sheath, and (3) TPI‐guided PFA.

The first step is the preparation for the visualization of the CS catheter and VIZIGO sheath, which is fundamental and crucial for the latter two steps. Once the RA matrix is created by the DECANAV catheter, nonmagnetic mapping catheters can be visualized along with the VIZIGO sheath. Therefore, fluoroscopy is not required for the placement of the CS catheter. In the second step, since the DECANAV catheter can be advanced to the superior vena cava, the VIZIGO sheath can be advanced over the DECANAV catheter in 3D‐EAM. Since the geometry of the FO was created, the transseptal puncture can be done easily by simply pulling back the VIZIGO sheath so that the tip of the VIZIGO overlaps the FO, as monitored by the ICE. Additionally, the VIZIGO sheath can be advanced in LA under 3D‐EAM guidance. This visualization helps avoid accidental perforation of the LA posterior wall caused by careless and blind advancement of the non‐visualized sheath. The third step would be the greatest advantage of the VARIPULSE‐based PFA. The VARIPULSE catheter can be manipulated in 3D‐EAM, and the catheter‐tissue contact can be confirmed with TPI, which does not require any fluoroscopy. In all patients, all PVIs were successfully performed using TPI‐guided PFA without fluoroscopy. Throughout the procedure, note that catheter‐cable switching is required multiple times due to the limited number of patient interface unit ports. In steps 1–2, the DECANAV mapping catheter is connected to port 20B. During the LA voltage/activation mapping after step 2, ports 20A and 20B are used for the OCTARAY catheter. During step 3, the VARIPULSE is connected to port 20A and the DECANAV catheter to port 20B for emergent ventricular pacing.

## FUNDING INFORMATION

N/A.

## CONFLICT OF INTEREST STATEMENT

Authors declare no conflict of interests for this article.

## ETHICS STATEMENT

Approval of research protocol: the study was approved by the Institutional Review Board of Nihon University Itabashi Hospital. Approval number: RK‐240312‐1. The research related to human use complied with all relevant national regulations and institutional policies and is in accordance with the tenets of the Helsinki Declaration.

## CONSENT

The patient has provided consent for publication.
